# Breast Cancer-Related Financial Toxicity in Sri Lanka: Insights From a Lower Middle-Income Country With Free Universal Public Healthcare

**DOI:** 10.1093/oncolo/oyad259

**Published:** 2023-09-22

**Authors:** Sarith Ranawaka, Sathika Gunarathna, Sanjeeva Gunasekera, Christopher M Booth, Matthew Jalink, Laura M Carson, Scott Berry, Bishal Gyawali, Sanjeewa Seneviratne, Don Thiwanka Wijeratne

**Affiliations:** Department of Surgery, Faculty of Medicine, University of Colombo, Colombo, Sri Lanka; Department of Surgery, Faculty of Medicine, University of Colombo, Colombo, Sri Lanka; National Cancer Institute, Maharagama, Sri Lanka; Department of Oncology, Queen’s University, Kingston, Ontario, Canada; Division of Cancer Care and Epidemiology, Queen’s University Cancer Research Institute, Kingston, Ontario, Canada; Division of Cancer Care and Epidemiology, Queen’s University Cancer Research Institute, Kingston, Ontario, Canada; Department of Public Health Sciences, Queen’s University, Kingston, Ontario, Canada; Division of Cancer Care and Epidemiology, Queen’s University Cancer Research Institute, Kingston, Ontario, Canada; Department of Oncology, Queen’s University, Kingston, Ontario, Canada; Department of Oncology, Queen’s University, Kingston, Ontario, Canada; Division of Cancer Care and Epidemiology, Queen’s University Cancer Research Institute, Kingston, Ontario, Canada; Department of Surgery, Faculty of Medicine, University of Colombo, Colombo, Sri Lanka; Division of Cancer Care and Epidemiology, Queen’s University Cancer Research Institute, Kingston, Ontario, Canada; Department of Medicine, Queen’s University, Kingston, Ontario, Canada

**Keywords:** Breast cancer, financial toxicity, lower-to-middle income countries, LMIC

## Abstract

Financial toxicity (FT) describes either objective or perceived excess financial strain due to a cancer diagnosis on the well-being of patients, families, and society. The consequences of FT have been shown to span countries of varied economic tiers and diverse healthcare models. This study attempts to describe FT and its effects in a lower- to middle-income country delivering predominantly public nonfee-levying healthcare. This was a cross-sectional study involving 210 patients with breast cancer of any stage (I to IV), interviewed between 6 and 18 months from the date of diagnosis. Financial toxicity was highly prevalent with 81% reporting 3 or more on a scale of 1 to 5. Costs incurred for travelling (94%), out-of-hospital investigations (87%), and consultation fees outside the public system (81%) were the most common contributors to FT. Daily compromises for food and education were made by 30% and 20%, respectively, with loss of work seen in over one-third. Greater FT was seen with advanced cancer stage and increasing distance to the nearest radiotherapy unit (*P* = .008 and .01, respectively). Family and relatives were the most common form of financial support (77.6%). In conclusion, FT is substantial in our group, with many having to make daily compromises for basic needs. Many opt to visit the fee-levying private sector for at least some part of their care, despite the availability of an established public nonfee-levying healthcare.

## Teaser

This article describes financial toxicity in a nonfee-levying healthcare system in South Asia to better enable care providers to be sensitive to the implications of their prescribed therapy and provide patient-centred care.

**Implications for Practice**

To our knowledge, this is the first study to describe financial toxicity in a nonfee-levying healthcare system in South Asia. These findings cater to knowledge gaps that would facilitate patient-centred interventions that are pragmatic and sustainable. This will enable care providers to be cognizant and sensitive to the implications of their prescribed therapy and, hence, optimize their practices toward patient-centred care. Through education, this study will also help modify healthcare healthcare-seeking behavior of patients visiting fee-levying private healthcare, despite the availability of public nonfee-levying healthcare.

## Introduction

Financial toxicity (FT) in cancer care, defined as “the detrimental effects of excess financial strain caused by a cancer diagnosis on the well-being of patients, their families and society,” is commonly caused by receipt of cancer therapy.^1-4^ FT encompasses both objective and perceived burden and is increasingly being acknowledged as an evolving clinical and social entity that can greatly affect the quality of life and survival of not just patients, but their families and society at large.^1,3-5^ These effects have been observed in high-income countries (HICs) with universal healthcare and/or health insurance and are expected to be even more pronounced in low- and middle-income countries (LMICs).^2,3,6^

FT with cancer results from a combination of medical expenses (investigations, hospital consultations, surgery, or adjuvant or neoadjuvant therapy), nonmedical expenses (travelling, lodging, food-related costs), and loss of income (loss of work by patient or family members).^7^ Many consequences have been observed, including patients forgoing or delaying treatment, resulting in an overall detriment in their quality of care and poorer survival.^2,8^ FT has also been reported to be a strong predictor of quality of life in patients with cancer.^9^ This is seen where some patients and families are required to compromise on basic daily needs such as food, clothing, and education activities.^3^

Financial hardships are strongly associated with national economic policies pertaining to healthcare provision.^4^ This helps explain the diversity in the burden of FT experienced by patients with cancer in different countries.^2^ Sri Lanka is a lower- to middle-income country (LMIC) with a unique dichotomy of nonfee-levying public healthcare catering to the majority of the population and fee-levying private healthcare, which is primarily insurance-based care used by a minority of its citizens.^10^ Due to resource limitations, overcrowding, perceived poor quality of medications, and longer wait times for some of the treatments and investigations, patients may seek part of their care outside the public health system through out-of-pocket expenditure. As of 2020, Sri Lanka’s gross domestic product (GDP) per capita and GDP per capita adjusted for purchasing power parity (GDP per capita, PPP) were USD 3682 and USD 13 255, respectively.^11^ This provides perspective on the impact of financial burden on patients.

Breast cancer is the most common cancer both in females and overall, in Sri Lanka.^12^ FT in this patient group has been widely reported globally.^13^ However, details of the FT experienced by Sri Lankan patients with breast cancer, including severity, consequences, and determinants are unknown. This is important as Sri Lanka is an LMIC with a predominantly publicly funded healthcare system. To our knowledge, FT in such a setting has not been described before. This study was conducted to identify, describe, and quantify FT in a cohort of women with breast cancer receiving treatment at the National Cancer Institute, Sri Lanka (NCISL), the premier public sector cancer care hospital in Sri Lanka.

## Methods

### Study Population

All women with newly diagnosed invasive primary breast cancers from January 01, 2016, to December 31, 2020, were identified from the NCISL breast cancer registry. The NCISL breast cancer registry is a prospectively maintained database of an inception cohort that includes all breast cancers treated at the NCISL from 2016 onwards.^14^ The NCISL is the largest dedicated cancer hospital in Sri Lanka, providing care for over one-third of the cancer population at some point in their cancer treatment.^14^ These patients are treated in a publicly funded healthcare system, in which nearly the entire cost of cancer therapy, most relevant investigations and pertinent healthcare services are funded by the public sector. Rarely, out-of-pocket specialized investigations and medications may be incurred by patients. Details on establishing the database, data capture and validation as well as the demography of patients with breast cancer included in this registry have been described previously.^14^ From this registry, those with breast cancer of any stage (I to IV) who were between 6 and 18 months post-date of diagnosis were eligible for inclusion. This time frame was selected as most women were nearing completion of treatment or had just completed adjuvant therapy, which would capture the maximum financial impact while minimizing recall bias. A random sample from those who were alive was selected for inclusion stratified by stage at diagnosis to ensure a sample representative of women included in the database. Patient demographic information relevant to these patients was extracted from the database.^14^

### Questionnaire Development and Administration

A questionnaire was developed to capture constructs relevant to patients with cancer in Sri Lanka. Previous questionnaires on FT used in other settings (i.e., Nepal, Italy) were used as templates to develop our questionnaire.^8,15^ A focus group composed of physicians, patients, and hospital administrators was formed to make adaptations that were relevant to the local population. Information on the type, magnitude, implications, barriers, and supports to overcome FT were used as the main constructs in developing this questionnaire. Table A1 for the full version of the questionnaire. As the income was variable, in addition to the actual monetary value, the financial impact that was perceived was quantified using a Likert scale of 1-5 depicting increasing financial burden with higher scores. The impact and magnitude of FT were further quantified in multiple socioeconomic domains including but not limited to loss of income, property, and loans.

Patients were contacted via phone by a trained interviewer. Where applicable, input from a caregiver was collected with the patient in their presence.

### Statistical Analysis

Counts and percentages were used to describe categorical and ordinal variables while mean, SD, median, and corresponding range were used to describe continuous variables depending on the variable’s distribution. The association between financial burden with available financial support, distance to NCISL and cancer stage were analyzed using Spearman’s test with a *P*-value of <.05 being considered as significant. Data analysis was performed using IBM Statistics Package Ver. 26 (SPSS).^16^

Ethics approval for this study was obtained from the Ethics Review Committee (EC-17-068) of the Faculty of Medicine, University of Colombo, Sri Lanka. Informed consent was obtained from each patient and their families prior interview of patients. Voluntary withdrawal at any point in the conduct of the study was offered to all patients. The de-identified data collected and analyzed in this study could be made available upon request.

## Results

### Patient Demographics

This study included 210 patients (approximately 1100 eligible patients). The mean age was 58 years (SD 11). Eighty-eight per cent (181/207) of participants were married, and 37% (51/138) were educated up to secondary school or higher (Table 1). Ninety per cent (188/210) had dependent children under 18 years of age. Most were diagnosed with stage II breast cancer (54%, 112/208) followed by stage III (26%, 54/208; Table 1). The mean (SD) duration from the date of diagnosis to the collection of data was 12 (4) months.

**Table 1.** Sociodemographic characteristics of patients with breast cancer.

Characteristic	*N* = 210 (%)
Marital status	
Married	181 (87)
Widowed	11 (5)
Divorced	3 (1)
Unmarried	12 (6)
Missing	3
Level of education	
Primary	52 (38)
Secondary	43 (31)
Graduate	8 (6)
None	35 (25)
Missing	72
Monthly income (Sri Lankan Rs.)	
<20 000	52 (38)
20 000-50 000	58 (43)
50 000-100 000	23 (17)
>100 000	3 (2)
Missing	74
Breast cancer stage	
I	26 (12)
II	112 (54)
III	54 (26)
IV	16 (8)
Missing	2

Of the patients who declared their monthly family income, most (81%, 110/136) had a monthly family income of less than 50 000 Sri Lankan Rupees (Rs.) or 250 USD with only 3 (2%) receiving a total family income of more than Rs.100 000 (500 USD) per month (Table 1).

The utilization of cancer care treatment resources by our cohort is presented in Table 2. Only a small number of patients (*n* = 22) received neoadjuvant care and only a third received surgical care. The majority of patients received radiotherapy and systemic treatment.

**Table 2.** Utilization of cancer care services at the National Cancer Institute of Sri Lanka by patients with breast cancer at the time of study.

Utilization of NCISL cancer care	*n* ^a^ (%)
Neoadjuvant therapy	22 (10.5)
Surgery	71 (33.8)^b^
Radiotherapy	128 (61)
Chemotherapy	138 (65.7)
Hormone therapy	116 (55.2)
Follow up care	175 (83.3)

^a^ *n* is more than the total population (210) given that the patients seek more than one form of cancer care during their visit.

^b^This includes only the women who had undergone surgery at the National Cancer Institute.

### Perceived Financial Toxicity

**Figure 1.**
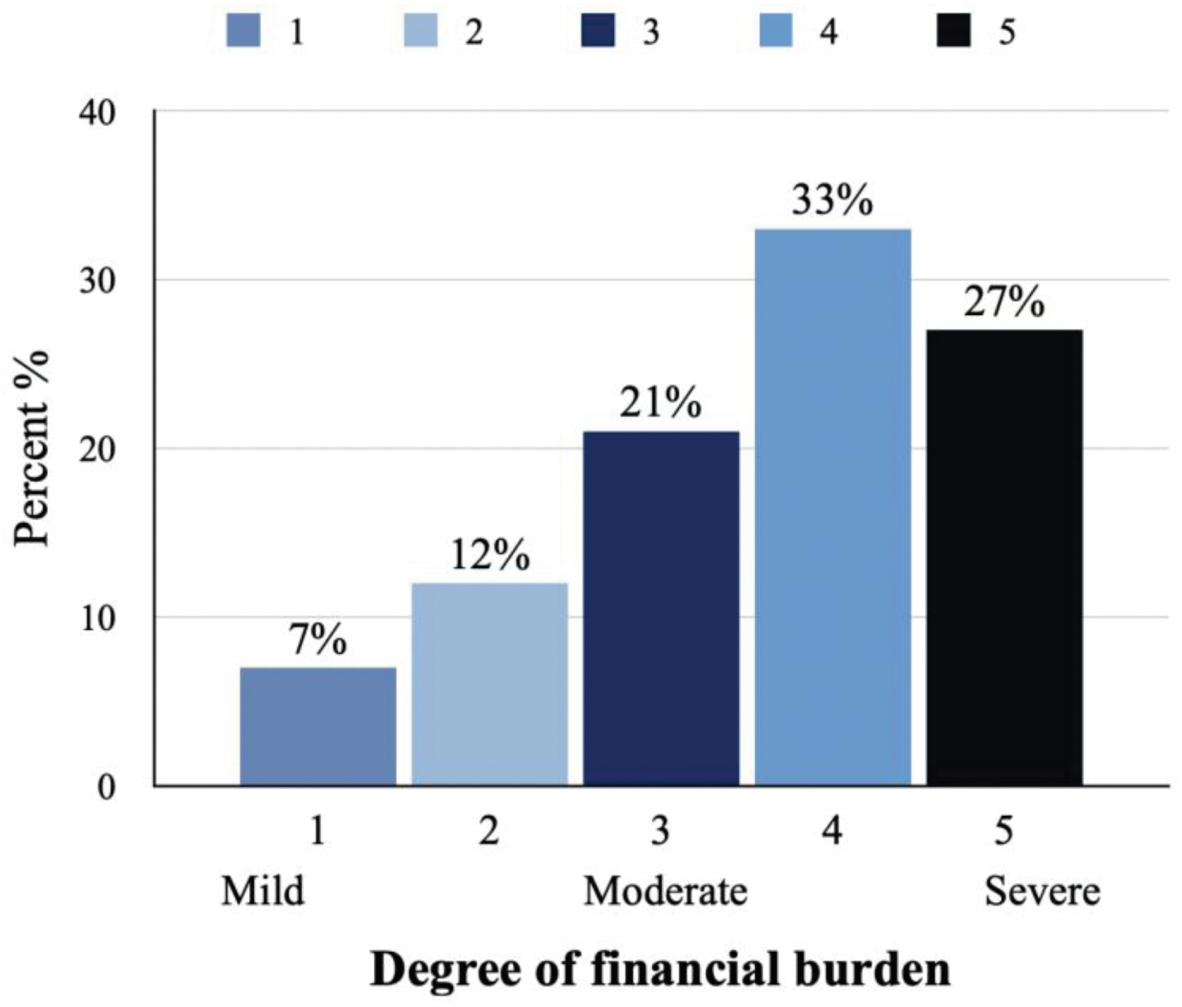
Perceived financial burden on a scale of 1-5 (1: minimal, 5 maximum toxicity).

A majority (81%, *n* = 169) reported at least a moderate perceived financial burden of 3 or more on a scale of 1-5 (1: minimum, 5: maximum; Fig. 1).

Median monthly out-of-pocket expense for treatment was Rs. 13 762 (70 USD) (mean = 27 000, 135 USD) and ranged between Rs. 265 and 300 000 (1-1500 USD). This mean per-month average out-of-pocket expenditure was greater than the monthly household earnings for 38% of patients.

Common reasons for out-of-pocket expenditure included travel (94%, 198/210), out-of-hospital investigations (laboratory and radiological) (87%, 183/210), and healthcare consultation fees (81%, 171/201), outside of the public system. Additional purchasing costs of cancer therapy that are not covered by public healthcare or unavailable and supportive care medication contributed to 39% (83/210) and 25% (53/210), respectively (Table 3).

**Table 3.** Contributors to financial toxicity based on perceived burden.

Characteristic	*N* (%)	Perceived burden *n* (%)				
		1	2	3	4	5
Travelling	198 (94)	14 (7)	23 (12)	43 (22)	67 (34)	51 (26)
Hospital investigations	183 (87)	15 (8)	22 (12)	41 (22)	62 (34)	43 (24)
Private consultations	171 (81)	13 (8)	20 (12)	35 (20)	57 (33)	46 (27)
Cancer treatment	83 (40)	8 (10)	9 (12)	23 (28)	23 (28)	20 (24)
Food	81 (39)	3 (4)	9 (11)	15 (19)	26 (32)	28 (35)
Supportive medication	53 (25)	3 (6)	3 (6)	12 (23)	19 (36)	16 (30)
Caregiver services	14 (7)	0	1 (7)	5 (36)	6 (43)	2 (14)
Lodging	3 (1)	0	1 (33)	0	1 (33)	1 (33)
Childcare services	3 (1)	0	0	0	2 (67)	1 (33)
Physiotherapy	2 (1)	1 (50)	0	0	0	1 (50)
Dental care	1 (0)	0	0	0	1 (100)	0

The median distance from the place of residence to the NCISL, the nearest satellite chemotherapy unit and the nearest radiotherapy unit were 38 km (range 1-200 km), 29 km (range 1-210 km), and 38 km (range 1-200 km), respectively.

### Impact of Financial Toxicity

The impacts of FT are shown in Table 4. Daily expenditure for food for the family was compromised in 30% (*n* = 63) participants, while 20% (*n* = 43) reported having to compromise on expenses for the education of their children/dependents (Table 4). Loss of work in the family and loss of property (either selling or pawning) were seen in 37% (*n* = 77) and 25% (*n* = 52) participants, respectively.

**Table 4.** Daily compromises made by study participants and their impact.

Characteristic	*N* (%)
Daily compromises made	
Food	63 (30)
Education	43 (20)
Holidaying	54 (26)
Recreation	39 (19)
Shelter	2 (1)
Impacts of financial toxicity	
Loss of work in the family	77 (37)
Loss of property (selling/pawning)	52 (25)
Having to resort to savings	96 (46)
Requiring private loans	27 (13)

The reasons for seeking private-sector consultations are shown in Table 5. The majority visited the private sector with the intention of seeking care from a preferred oncologist (49%, *n* = 102) while 35% (*n* = 73) had sought private healthcare with the perception of “shorter” waiting times.

**Table 5.** Reasons for seeking private sector consultations.

Reasons for seeking private sector consultations	*n* (%)
To meet the preferred oncologist	102 (49)
Perceived shorter waiting times for consultation	73 (35)
To obtain additional information	52 (25)
To reduce COVID-related risks	37 (18)
For relatives to meet the oncologist	28 (13)
To obtain “better” care	13 (6)

### Financial Toxicity by Stratified Demographics

Greater financial burden was associated with advanced cancer stage (correlation coefficient ϼ=0.18, *P* = .008) and with increasing distance to the nearest radiotherapy unit (ϼ=0.17, *P* = .01). The financial burden was also more pronounced in families with low family income (ϼ=−0.22, *P* = .01). There was no significant difference in the rates of private health sector utilization between different income groups (*P* = .33).

No significant associations were observed between severity of toxicity and factors that contributed to financial toxicity (*P* > .05). Furthermore, there was no statistically significant association between patients seeking care in the private sector with cancer stage (*P* = .224), marital status (*P* = .689), monthly income (*P* = .156), or level of education (*P* = .327). There was also no relationship between care in the private sector and distance to NCISL (*t* = −1.02, *P* = .309).

Sources of financial support for patients and/or families are shown in Table 6. Some form of financial support was available to 86% (181/210) of participants. The predominant means of support was from other family members/relatives (77%, 163/210), followed by financial aid from the government (20%, 41/210) and members of their respective communities (13%, 28/210). Only a small minority (11%, 24/210) benefitted from healthcare insurance (Table 3).

**Table 6.** Sources of financial support available.

Sources of financial support available	*n* (%)
Family/relatives	163 (77.6)
Government aid	41 (19.5)
Community	28 (13.3)
Health insurance	24 (11.4)
Workplace	24 (11.4)
Fundraisers	10 (4.8)
Hospital	1 (0.5)

## Discussion

To our knowledge, this is the first study conducted in Sri Lanka that attempts to describe FT in patients with breast cancer. This is especially important, as this study assesses the financial impact in a publicly funded healthcare system which to our knowledge has not been studied in South Asia before. This study was conducted before the current financial crisis in Sri Lanka; hence, the findings are reflective of usual cancer care. Breast cancer in women has a significant impact on families and communities.^17^ This study highlights the substantial financial burden among the majority of Sri Lankan women with breast cancer. Such a high prevalence of FT was observed despite the cohort being selected from a public healthcare facility in a country that has nonfee-levying universal healthcare.

Many studies have shown low monthly household income and advanced cancer stage to be associated with greater financial burden.^1,3,18^ The results from our study are consistent with these findings. Furthermore, a significant FT was seen across all stages of breast cancer with a high correlation with cancer stage.

Almost all participants had nontreatment-related expenses contributing to out-of-pocket expenditures. Expenses for travelling were borne by almost all, which was a result of patients having to travel a median of 17 to 25 km (34 to 50 km both ways) to reach their nearest satellite chemotherapy or radiotherapy unit. In addition, some patients required visiting different hospitals, sometimes multiple times per month for treatment. High cost of travel has been shown to intensify financial burden,^18^ as previously reported by Lentz et al.^1^ Arranging cost reimbursements for travelling to those requiring therapy (especially radiation) likely would have a significant impact on alleviating its contribution to FT. This is already being done for patients with childhood cancer at NCISL, indicating a potential opportunity for scale-up to the adult population. Similarly, financial equity programs have been implemented in cancer clinical trial enrolment and have shown increased accrual following reimbursements for travelling and lodging.^19^

The current scarcity of radiation therapy units^20^ is a potential explanation for travelling costs, demonstrated by a significant rise in expenses with increasing distance to the nearest radiation therapy unit. Despite the widespread availability of both public and private centres for surgery in the country, radiation therapy facilities are limited to 7 hospitals.^21^ At the time of this study, there were 7 (5 public and 2 private) functioning linear accelerators distributed in 4 of 9 provinces, with plans to implement a total of 12 (10 public and 2 private) in 5 provinces in the near future.^22^ This may reduce not only the travel distance but also the long waiting times experienced by some patients.

Despite obtaining treatment from a free-public sector hospital, 81% had consulted a doctor in a private hospital with 87% having undergone medical investigations in a private hospital. Income level was not associated with the decision to utilize private healthcare (consultation/investigations/active treatment for cancer). Given that most doctors who practice in the private sector are employed at government hospitals as their primary location of work, the exact reasons for such consultations need further exploration. However, this highlights potential improvements that could be achieved through streamlining patient referral and educating patients on the availability and efficiency of cancer care services in the public sector.

Loss of work in the family was seen in over one-third of the patients. One-fourth of the patients had to either pawn/sell property while others resorted to private loans. These results can be contrasted to those of a similar study in Malaysia, an LMIC with universal access to healthcare, in which only 5% of patients with cancer incurred a significant financial burden.^18^ Bhoo-Pathy et al also reported only 25% of a cohort of 1662 patients in Malaysia with newly diagnosed cancer had to resort to using lifelong savings to meet expenses, compared to almost 50% of our study group (46%).^18^ In addition, many participants and their families in our study had to compromise on basic daily needs such as food and logistical costs for education. A considerable proportion in our cohort was also unable to continue with leisurely recreation and holiday activities, which have been shown to contribute adversely toward their Quality of life (QOL).^3^

Several studies conducted in other publicly funded healthcare systems in high-income countries (HICs) have shown high levels of FT among patients with cancer. For example, a study exploring FT in Japan found that 60% of surveyed patients used alternative strategies, such as reducing spending on food or clothing, to manage FT. Similar findings have been reported in other high-income countries with publicly funded healthcare systems such as Italy.^8,23^ Those conducted in other South Asian LMICs without a publicly funded healthcare system have shown even more severe levels of FT. A survey of patients with acute leukaemia in Nepal found that 73% of respondents were classified as enduring “extreme FT,” defined as having to sell property to support treatment, taking a monetary interest-based loan from others and fundraising or asking for charity from the public. Our study highlights the persistence of FT despite a predominately publicly funded healthcare system, which is alarming.

Further exploration of likely sociodemographic and psychosocial attributes that may be specific to this population and contribute to such vulnerability should be further explored. Possible explanations may include patient perception and limited knowledge of the availability and quality of care provision available in the public sector. Other contributors may include patient preference for seeking care in more central tertiary care centers, and family and societal expectations, and values around prioritizing the best possible perceived care. These are likely intertwined with the diverse cultural and religious values of patients and in wider society in Sri Lanka.

The most commonly reported risk factors for greater FT for women with breast cancer include female gender, younger age, a low income at baseline, receiving adjuvant therapies, a recent diagnosis, and advanced stage of cancer.^3,6^ Access to private insurance or private healthcare does not make one immune to FT of cancer, as many other factors including loss of income of the individual as well as other family members, costs of travel, accommodation, and other supplementary medical care that are not covered with most insurance coverages can collectively contribute.^2^

Even though over 3 quarters of patients resorted to some form of financial assistance, it is difficult to quantify the extent of this support. The majority were dependent on extended family and relatives for financial support, a finding that is unique to our study population. This form of support is rarely the most common form of support in other countries, where the rates are as low as 15%.^3,18^ Close relationships with extended family and cultural beliefs in helping the sick may contribute to these observations.

Most countries have adopted formal financial support systems to aid patients with cancer. This may exist in the form of government/workplace funds or through healthcare-related payment allowances, such as transportation, cost reimbursement, etc. Sri Lankan government currently provides a monthly nominal amount of 3000 Sri Lankan Rupees (LKR) (approximately 5% of average monthly household income) to those diagnosed with cancer who have a monthly household income LKR less than 6000 (<10% of average monthly household income).^24^ Patients may also be entitled to small amounts of additional financial aid through the government if disabled or facing other social hardships.

Several studies suggest the importance of screening for patients at high risk of FT, pretreatment cost disclosure by hospitals and better doctor-patient communication early in the management of patients with cancer as potential strategies for mitigating FT.^2-4,6^ Although cost disclosure is an important step in the fee-levying/primarily insurance-based setting, greater vigilance toward potential nontreatment-related costs (especially cost for travel) may be important in reducing FT in the public nonfee-levying healthcare system. Several scoring systems and shared decision-making tools have been developed to better aid this conversation between the patient and physician. These include the American Society of Clinical Oncology (ASCO) value framework and magnitude of clinical benefit scale (MCBS).^4^

We acknowledge several limitations in this study. First, the study was done with a relatively small sample of women receiving treatment from a single tertiary care hospital. There was nondisclosure of some demographic information by a minority of patients. Therefore, the findings may not reflect the true FT among all women with breast cancer in Sri Lanka, although our sample was representative of a large catchment area of the country. Furthermore, there is a potential for recall bias as data were collected based on average expenditure during the preceding 6 months. In addition, the perceived reasons for toxicity and reasons for seeking care from the private sector remain to be explored further.

## Conclusion

FT observed to be substantial in our cohort of patients may be attributable to current policy commitment by the government to a public model, and perhaps in part because of insufficient public resources or shortcomings with implementing robust nonfee levying care. The recent financial downturn the country has experienced would likely have further exacerbated FT due to both reduced availability of medicines and investigations through the public sector and reduced individual affordability. Perhaps, the most important inference that is easily modifiable from our study is that many patients with breast cancer visit the fee-levying private sector for at least some part of their care, despite the “availability” of public nonfee-levying healthcare. This study presents a preliminary effort to acknowledge and quantify FT in cancer care in Sri Lanka, which is a significant stepping stone warranting further exploration and subsequent action.

## Data Availability

The de-identified data collected and analyzed in this study could be made available upon request to the corresponding author.

## Appendix

**Table A1.** Mean burden scores (out of 5) and *P*-values for each contributor to out-of-pocket expenses.

Contributors to out-of-pocket expenses	Mean burden experienced out of 5 (SD)		p value (*t-test*)
	Yes	No	
Travelling	3.6 (1.2)	3.5 (1.6)	0.844
Hospital investigations	3.5 (1.2)	4.0 (1.2)	***0.041**
Private consultation charges	3.6 (1.2)	3.5 (1.2)	0.768
Active management of cancer (surgery/chemotherapy/radiotherapy)	3.5 (1.2)	3.7 (1.2)	0.202
Food	3.8 (1.1)	3.4 (1.2)	***0.025**
Supportive medication	3.8 (1.1)	3.5 (1.2)	0.162
Caregiver services	3.6 (0.8)	3.6 (1.2)	0.868
Lodging	3.7 (1.5)	3.6 (1.2)	0.913
Childcare services	4.3 (0.6)	3.6 (1.2)	0.287
Physiotherapy	3.0 (2.9)	3.6 (1.2)	0.816
Dental care	4.0 (4.0)	3.6 (1.2)	0.736

## References

[1] Lentz R , Benson AB, Kircher S. Financial toxicity in cancer care: prevalence, causes, consequences, and reduction strategies. J Surg Oncol. 2019;120(1):85-92. 10.1002/jso.25374 30650186

[2] Gordon LG , Merollini KMD, Lowe A, Chan RJ. A systematic review of financial toxicity among cancer survivors: we can’t pay the co-pay. Patient. 2017;10(3):295-309. 10.1007/s40271-016-0204-x 27798816

[3] Rosenzweig M , West M, Matthews J, et al. Financial toxicity among women with metastatic breast cancer. Oncol Nurs Forum. 2019;46(1):83-91. 30547962 10.1188/19.ONF.83-91

[4] Desai A , Gyawali B. Financial toxicity of cancer treatment: moving the discussion from acknowledgement of the problem to identifying solutions. EClinicalMedicine. 2020;20:100269. 10.1016/j.eclinm.2020.100269. 32300733 PMC7152810

[5] Souza JA de , Yap BJ, Wroblewski K, et al. Measuring financial toxicity as a clinically relevant patient-reported outcome: the validation of the COmprehensive Score for financial Toxicity (COST). Cancer. 2017;123(3):476-484. 27716900 10.1002/cncr.30369 PMC5298039

[6] Jagsi R , Ward KC, Abrahamse PH, et al. Unmet need for clinician engagement regarding financial toxicity after diagnosis of breast cancer. Cancer. 2018;124(18):3668-3676. 10.1002/cncr.31532 30033631 PMC6553459

[7] Witte J , Mehlis K, Surmann B, et al. Methods for measuring financial toxicity after cancer diagnosis and treatment: a systematic review and its implications. Ann Oncol. 2019;30(7):1061-1070. 10.1093/annonc/mdz140 31046080 PMC6637374

[8] Perrone F , Jommi C, Maio MD, et al. The association of financial difficulties with clinical outcomes in cancer patients: secondary analysis of 16 academic prospective clinical trials conducted in Italy. Ann Oncol. 2016;27(12):2224-2229. 27789469 10.1093/annonc/mdw433

[9] Fenn KM , Evans SB, McCorkle R, et al. Impact of financial burden of cancer on survivors’ quality of life. J Oncol Pract. 2014;10(5):332-338. 10.1200/JOP.2013.001322 24865220

[10] New World Bank country classifications by income level: 2022-2023. 2022. Available from https://blogs.worldbank.org/opendata/new-world-bank-country-classifications-income-level-2022-2023

[11] GDP per capita (current US$) - Sri Lanka | Data. Available from: https://data.worldbank.org/indicator/NY.GDP.PCAP.CD?locations=LK

[12] Worldwide cancer data | World Cancer Research Fund International. WCRF International. Available from: https://www.wcrf.org/dietandcancer/worldwide-cancer-data/

[13] Gharzai LA , Ryan KA, Szczygiel L, et al. Financial toxicity during breast cancer treatment: a qualitative analysis to inform strategies for mitigation. JCO Oncol Pract. 2021;17(10):e1413-e1423. 10.1200/OP.21.00182 34251880

[14] Wijeratne DT , Gunasekera S, Booth CM, Promod H, Seneviratne S. Patterns of cancer care in Sri Lanka: assessing care provision and unmet needs through an electronic database. J Cancer Policy. 2020;25:100243. 10.1016/j.jcpo.2020.100243

[15] Poudyal BS , Giri S, Tuladhar S, Neupane S, Gyawali B. A survey in Nepalese patients with acute leukaemia: a starting point for defining financial toxicity of cancer care in low-income and middle-income countries. Lancet Haematol. 2020;7(9):e638-e639. 10.1016/S2352-3026(20)30258-1 32853583

[16] SPSS Statistics | IBM [Internet]. Available from: https://www.ibm.com/products/spss-statistics

[17] Keesing S , Rosenwax L, McNamara B. The implications of women’s activity limitations and role disruptions during breast cancer survivorship. Women’s Health. 2018;14:174550571875638. 10.1177/1745505718756381 PMC5808959 29409399

[18] Bhoo-Pathy N , Ng CW, Lim GCC, et al. Financial toxicity after cancer in a setting with universal health coverage: a call for urgent action. J Oncol Pract. 2019;15(6):e537-e546. 10.1200/JOP.18.00619 31112479

[19] Nipp RD , Lee H, Powell E, Birrer NE, Poles E, Finkelstein D, et al. Financial Burden of Cancer Clinical Trial Participation and the Impact of a Cancer Care Equity Program. Available from: https://theoncologist.onlinelibrary.wiley.com/doi/10.1634/theoncologist.2015-0481 10.1634/theoncologist.2015-0481 PMC4828126 26975867

[20] Gunasekera S , Seneviratne S, Wijeratne T, Booth CM. Delivery of cancer care in Sri Lanka. J Cancer Policy. 2018;18:20-24.

[21] Jayarajah U , Abeygunasekera AM. Cancer services in Sri Lanka: current status and future directions. J Egypt Natl Canc Inst. 2021;33(1):13. 10.1186/s43046-021-00070-8 34081229 PMC13316883

[22] Joseph N , Gunasekera S, Ariyaratne Y, Choudhury A. Clinical oncology in Sri Lanka: embracing the promise of the future. Int J Radiat Oncol Biol Phys. 2019;105(3):466-470. 10.1016/j.ijrobp.2019.04.023 31540589

[23] Honda K , Gyawali B, Ando M, et al. Prospective survey of financial toxicity measured by the comprehensive score for financial toxicity in Japanese patients with cancer. J Global Oncol. 2019;5(5):1-8. 10.1200/JGO.19.00003 PMC6550026 31070981

[24] Household Income and Expenditure Survey-2016 Final Results. Department Of Census And Statistics, Ministry of National Policies and Economic Affairs, Colombo, Sri Lanka 2017. ISSN-2012-760X

